# *Metarhizium brunneum* (Hypocreales: Clavicipitaceae) and Its Derived Volatile Organic Compounds as Biostimulants of Commercially Valuable Angiosperms and Gymnosperms

**DOI:** 10.3390/jof8101052

**Published:** 2022-10-08

**Authors:** Martyn J. Wood, Alexandra M. Kortsinoglou, Salim Khoja, Vassili N. Kouvelis, Arben Myrta, Audun Midthassel, E. Joel Loveridge, Tariq M. Butt

**Affiliations:** 1 Department of Biosciences, Swansea University, Singleton Park, Swansea SA2 8PP, UK; 2 Department of Biology, Section of Genetics and Biotechnology, National and Kapodistrian University of Athens, 157 72 Athens, Greece; 3 CERTIS BELCHIM BV, R&D Department, 3521 AZ Utrecht, The Netherlands

**Keywords:** volatile organic compounds, entomopathogenic fungi, *Metarhizium brunneum*, biostimulation, hydrogels, plants

## Abstract

*Metarhizium brunneum* is a highly effective entomopathogenic fungus that also functions as a plant biostimulant. It can act as both an endophyte and rhizosphere colonizer; however, the mechanisms driving biostimulation are multifactorial. In this work, oilseed rape (*Brassica napus*) seeds were grown in composts treated with different concentrations of *M. brunneum* strains ARSEF 4556 or V275, or the *M. brunneum*-derived volatile organic compounds 1-octen-3-ol and 3-octanone. Biostimulation efficacy was found to be strongly dose dependent. Concentrations of 1 × 10^6^ conidia g^−1^ compost were found to be most effective for the *M. brunneum*, whereas dosages of 1 µL 100 g^−1^ compost were found to be efficacious for the volatiles. These optimized doses were assessed individually and in combined formulations with a hydrogel against oilseed rape (*Brassica napus*), sitka spruce (*Picea sitchensis*), maize (*Zea mays*) and strawberry (*Fragaria annanassa*). Both volatile compounds were highly effective biostimulants and were found to increase in biostimulatory efficiency when combined with *M. brunneum* conidia. Hydrogels were not found to interact with the growth process and may offer avenues for novel formulation technologies. This study demonstrates that *Metarhizium*-derived volatile organic compounds are actively involved in plant growth promotion and have potential for use in novel formulations to increase the growth of a wide range of commercially relevant crops.

## 1. Introduction

Entomopathogenic fungi (EPF) belonging to the genera *Beauveria* and *Metarhizium* are widely distributed pathogens of insects, mites and ticks [1]. Most attention to date has focused on exploiting EPF as Biological Control Agents (BCAs) because they offer an environmentally friendly alternative to conventional chemical pesticides [2]. There is, however, increasing evidence that EPF can also stimulate plant growth as endophytes or rhizosphere colonizers. Different strains of these fungi have been recovered from the rhizospheres and plant tissues of a wide range of wild and cultivated plant species [3], including many economically important crops such as cotton [4], tomatoes [5] and beans [6]. Some studies have also reported the presence of EPF as endophytes in gymnosperms, but no assessment for plant growth promotion was made [7,8,9]. In this state, endophytic EPF offer various beneficial advantages to their plant hosts, as they promote plant growth, provide resistance to abiotic stress and protect their host from insects and other phytopathogenic microbes [10,11,12,13,14,15,16,17]. 

Among the mechanisms that contribute to the above-mentioned endophytic mode of action by EPF, secondary metabolism and its components are key factors. Similar to other endophytes, EPF have a large metabolic repertoire and are capable of producing numerous secondary metabolites, including antibiotic compounds, Volatile Organic Compounds (VOCs), insecticidal enzymes and cytotoxic actives [18,19,20,21]. Studies propose that endophytic EPF regulate plants’ secondary metabolism, enhancing the production of compounds necessary for plant development [22,23,24]. In addition, *Metarhizium* species also produce indole-3-acetic acid (IAA), a hormone typically produced by plants, which plays a pivotal role in plant cell elongation, division and differentiation, and also appears to influence the growth and virulence of *Metarhizium* and other fungi [25]. 

Microbial VOCs produced by soil and plant-related microorganisms are involved in various types of intra- or inter-kingdom interactions, including microbe–microbe, microbe–plant, plant–insect and microbe–insect interactions [26,27,28,29,30]. Interactions mediated by microbial VOCs may occur above ground and below ground, while their emission can be unidirectional or bidirectional, as a chemical dialogue between interacting hosts [31,32]. Some case studies have already shown that several microbial VOCs may stimulate growth of plants such as *Arabidopsis* and tobacco [33,34]. Most of these studies, however, refer to VOC emission by bacteria; of those relating to fungi, most have been *in vitro* [34]. Despite extensive studies into microbial VOC interactions within the wider environment, up to now, little work has been conducted to determine the effect of endophytic entomopathogenic VOC emissions on plant growth activities.

Despite the well-described benefits that endophytic EPF provide to plant hosts, several factors can influence their efficacy as plant colonizers. The various physiological attributes of different plant species, the methods of artificial inoculation and the unstable environmental conditions are factors that determine the success of inoculation, the stability and the growth promoting activities of these fungi [14]. For this reason, formulation techniques play a major role in the evaluation of endophytic EPF strains as effective biofertilizers or biopesticides. Hydrogels are synthetic hydrophilic polymers that can absorb and retain large quantities of liquid [35,36]. For this reason, their usage has been proposed in horticulture to increase the water holding capacity of soil, especially in harsh environmental conditions such as drought periods, thereby increasing plant survival and growth [37]. Due to their retention properties, however, they have also been tested as slow-release pesticides and fertilizers [38,39]. This may be very useful in field conditions, as it increases the presence of these compounds in the soil and subsequently their action time duration, alleviating the problems related to the successful application of EPF, such as the changing environmental conditions. 

This study investigates the application effects of (a) two EPF strains, already well established as biopesticides, and (b) two of the VOCs they produce in promoting plant growth. A preliminary screening of VOCs identified as constituents in *M. brunneum* volatile bouquets identified 1-octen-3-ol and 3-octanone as the most effective biostimulants of those screened (data not published). These two compounds were selected for assay in the present series of assays. In addition, the degree to which the hydrogels may alter the effects of the application of fungi and VOCs combined on plant growth is also assessed. Herein is presented the first evidence that volatiles of the entomopathogenic fungus *Metarhizium brunneum* are able to stimulate plant growth independent of the fungus, while the addition of hydrogels was not found to impact on plant growth in any of the treatments, making it a suitable avenue of investigation for novel formulation technologies.

## 2. Materials and Methods

### 2.1. Metarhizium Production

*Metarhizium brunneum* strains V275 (commercial name: Met52) and ARSEF 4556 were obtained from the USDA ARSEF culture collection and maintained on Sabouraud Dextrose Agar (SDA) plates. Aerial conidia were mass-produced on broken basmati rice as described in Ansari and Butt [40], air-dried and subsequently harvested using a Mycoharvester (Mycoharvester, UK). All fungal cultures and spore stock were stored at 4 °C ± 1 °C for up to a month prior to experimental usage.

### 2.2. Assays to Determine Optimum M. brunneum Dosage

Initial assays were performed to determine the optimal concentrations for plant growth promotion of *M. brunneum*. Multi-purpose compost containing John Innes (Westfield, UK) was air dried in glasshouses maintained at 25 ± 2 °C for 5 days prior to experimentation. Conidia g^−1^ of *M. brunnneum* strains ARSEF 4556 and V275 were calculated using a modified Neubauer haemocytometer and subsequent calculations were made to apply concentrations of 1 × 10^5^, 1 × 10^6^, 1 × 10^7^ and 1 × 10^8^ conidia per gram of dry compost. In total, 200 g of the conidia-compost mix were added to 1 L square pots, and the soil was wetted to reach 60 ± 5% soil RH. 

Oilseed rape (OSR) (*Brassica napus* [Brassicales: Brassicaceae], Puffin Produce) seeds were placed in a 90 mm Petri dish containing a Whattman grade 1 filter paper soaked with 2 mL water, sealed with parafilm and incubated for 24 h at 24 ± 1 °C. Resultant germinated seedlings were selected, and into each prepared 0.75 L square pot (80 × 80 × 120 mm) a divot was made 10 mm deep before a seedling was transplanted and re-covered with compost. Plants were grown in a glasshouse maintained at 25 ± 2 °C. After six weeks, plants were removed from their pots and excess soil washed off, before the plants were dried indoors on absorbent paper for 1 h at 22 ± 2 °C. Subsequently, fresh weight measurements for each whole plant were recorded. Control experiments were grown in manufacturer compost without additional treatment. Five plants were used per replicate, with three technical replicates per experiment. The whole experiment was repeated twice (*n* = 30).

### 2.3. Assays to Determine Optimum VOC Dosage

Similarly, initial assays were performed to determine the optimal concentration of *Metarhizium*-derived VOCs for plant growth promotion of *M. brunneum*. Two key *Metarhizium*-derived VOCs [30]—3-octanone (Sigma-Aldrich, Gillingham, UK, 106-68-3, ≥98%) and 1-octen-3-ol (Sigma-Aldrich, Gillingham, UK 3391-86-4, ≥98%)—were prepared for assay using air-dried compost containing John Innes (Westfield, UK). Dried compost was mixed with either of the VOCs at a rate of 0.01, 0.1, 1 or 10 µL per 100 g compost. Compost was mixed thoroughly with the VOCs and wetted to 60 ± 5 % soil RH. Oilseed rape seeds were placed in Petri dishes to germinate as described in Section 2.1. Resultant germinated seedlings were selected, and into each prepared 0.75 L square pots (80 × 80 × 120 mm) pot a divot was made 10 mm deep before a seedling was transplanted and re-covered with compost. Plants were grown in a glasshouse as previously described, and fresh weights were recorded. Control experiments were grown in manufacturer compost without additional treatment. Five plants were used per replicate, with three technical replicates per experiment. The whole experiment was repeated twice (*n* = 30).

### 2.4. Effects of the Optimized Doses of M. brunneum, VOCs and Hydrogels on Four Commercial Plants

Follow-on experiment using the optimum dosage of each of the *M. brunneum* strains ARSEF 4556 and V275, the optimized dosage of each VOC and a commercial hydrogel (MiracleGro, UK), were performed to assess efficacy across a range of commercial crops in greenhouse assays. Sitka spruce (*Picea sitchensis* [Pinales: Pinaceae], Maelor Forest Industries, Whitchurch, UK), OSR (*Brassica napus*, Puffin Produce, Haverfordwest, UK), maize (*Zea mays* [Poales: Poaceae]) and strawberry (*Fragaria annanassa* [Rosales: Rosaceae], WB Chambers Ltd., Maidstone, UK) plants were chosen to assess the efficacy of the biostimulatory formulations, representing a range of commercially important species comprising both angiosperms and gymnosperms. Maize and OSR plants were grown from seed; seeds of each were prepared in a 90 mm Petri dish containing a Whattman grade 1 filter paper soaked with 2 mL water, sealed with parafilm and incubated for 24 h at 24 ± 1 °C. Seed which had begun to germinate after 24 h were selected for the follow-on experiments. Spruce and strawberry plants were obtained as plugs; therefore, initial plug weights were checked for variance prior to assay, and no differences were detected between means of plants contained within treatment groups prior to assay. 

Multi-purpose compost with John Innes (Westfield, UK) was air dried prior to experimentation and treatments prepared as in Section 2.1. For spruce, OSR and maize, 0.75 L square pots (80 × 80 × 120 mm) filled with 200 g dry soil preparation were used. Due to the size of strawberry plants, 2 L pots were used, containing 500 g of prepared compost. *Metarhizium* treatments were prepared using 1 × 10^6^ conidia g^−1^ compost and VOC treatments were prepared using 1 µL VOC per 100 g of compost. Potassium polyacrylate hydrogel crystals (Miracle Gro, Cardiff, UK) were selected for compatibility assessment and were prepared according to manufacturer instructions at a rate of 2.5 g per 100 g compost. Hydrogel and *Metarhizium* treatments were mixed into the dry compost first to ensure even spread prior to wetting. All materials were bought or produced less than one week prior to the start of assay. Each treatment was assayed individually and as a combination of all three.

Soil preparations were transferred to their pots, and a divot made in the centre of each. Germinated seedlings or plant plugs were transferred into the divot and re-covered with soil. OSR, maize and strawberry plants were left to grow in a glasshouse maintained at 25 ± 2 °C for six weeks. Sitka spruce plants were grown under the same conditions but for an extended period of 12 weeks. At the end point of the experiment, plants were removed from their pots and soil washed from the roots before being left to air dry indoors at 21 ± 2 °C for 1 h. Whole plants were then weighed before the shoot and root portions of the plant were separated for individual weighing. Control treatments were grown in multi-purpose compost only. Five plants were used per replicate, with three technical replicates per experiment. The whole experiment was repeated twice (*n* = 30).

### 2.5. Statistical Analysis

For OSR, strawberry and maize, measured end-point biomass was used directly for analysis. Given the high variability in spruce plugs, initial weights were recorded for each plant. Cursory statistical analysis between treatment groupings confirmed that there were no significant differences between groups prior to experimentation; therefore, end-point metrics were used for all analysis for spruce places also.

All experiments were analysed with ANOVA with Tukey’s post hoc test for multiple comparisons. These were performed using SPSS v28 (IBM corporation, Armonk, NY, USA). All graphs were created using Prism Graphpad v.8.4.3 (San Diego, CA, USA). 

## 3. Results

### 3.1. Dose Dependent Effects of Metarhizium brunneum Applied to Oilseed Rape

**Figure 1:**
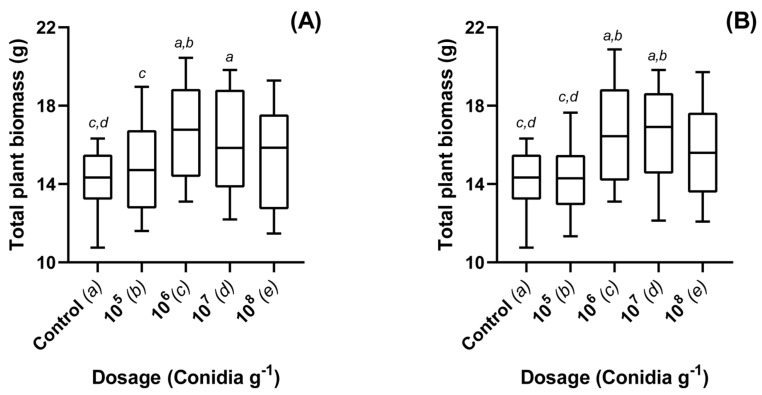
Total plant biomass (g) of Oilseed rape plants treated with different concentrations of *Metarhizium brunneum* strains: (**A**) V275 and (**B**) 4556. Whole plant fresh weights recorded for OSR exposed to different concentrations V275 or ARSEF 4556 as conidia g^−1^ of compost. Plants were grown in a glasshouse maintained at 25 ± 2 °C for a total of six weeks. Controls were exposed to compost media without additional treatment. Figure display’s minimum, 1st quartile, median, 3rd quartile and maximum measurements within the treatment group. Significant results denoted between treatments using letters (*a*–*e*) above each boxplot; where denotation letters are absent, no significant differences were detected between treatments.

Both *M. brunneum* strains ARSEF 4556 and V275 were found to elicit significant biostimulatory effects in OSR plants (*p* < 0.001). Growth was found to be dose dependent (Figure 1A). Composts treated at a rate of 1 × 10^5^ conidia g^−1^ were not found to significantly improve growth for either ARSEF 4556 (*p* = 1) or V275 (*p* = 0.991). Likewise, at the highest concentration (1 × 10^8^ conidia g^−1^), neither of the strains were found to cause significant effects. Treatments of 1 × 10^7^ conidia g^−1^ were effective using both strains (*p* = 0.001 and 0.026, respectively), where fresh weight was found to be 13.5–17.5% greater than controls over six weeks (Figure 1). Maximal fresh weight gains were found for treatments of 1 × 10^6^ conidia g^−1^. For V275, this was the most effective treatment as compared to controls (*p* < 0.001) (Figure 1A). For ARSEF 4556, this treatment also caused significant growth; however, there was no significant difference between this and either the 1 × 10^7^ or 1 × 10^8^ conidia g^−1^ treatments (Figure 1B).

### 3.2. Dose Dependent Effects of Metarhizium-VOCs Applied to Oilseed Rape

**Figure 2:**
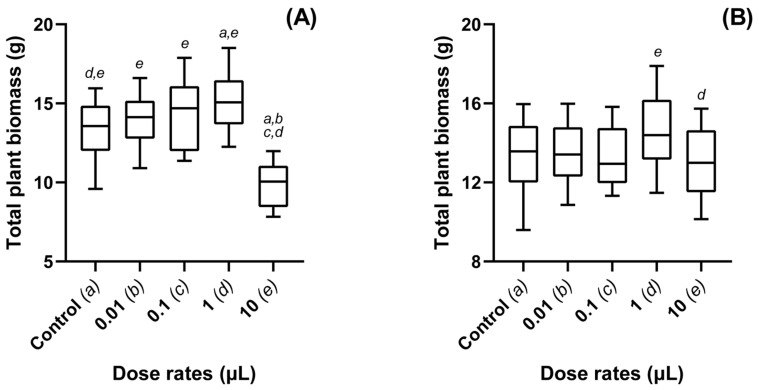
Total plant biomass (g) of Oilseed rape plants treated with different dosages of the *Metarhizium brunneum*-derived VOCs: (**A**) 1-octen-3-ol and (**B**) 3-octanone. Whole plant fresh weights recorded for OSR exposed to different concentrations of 1-octen-3-ol and 3-octanone. Dosage rates are represented as µL per 100 g dry compost. Plants were grown in a glasshouse maintained at 25 ± 2 °C for a total of six weeks. Controls were exposed to compost media without additional treatment. Figure display’s minimum, 1st quartile, median, 3rd quartile and maximum measurements within the treatment group. Significant results denoted between treatments using letters (*a*–*e*) above each boxplot; where denotation letters are absent, no significant differences were detected between treatments.

1-octen-3-ol was found to significantly improve plant growth, with dose dependency being a key factor in growth response (*p* < 0.001) (Figure 2A). In the highest 10 µL per 100 g dose of 1-octen-3-ol, several plants (*n* = 10) failed to germinate, suggesting strong phytotoxic effects at such high doses. No statistical differences were found between controls and plants treated with 1-octen-3-ol at a rate of 0.01 or 0.1 µL per 100 g. High stimulatory efficacy was, however, found at a dosage of 1 µL per 100 g (*p* < 0.001).

In the case of 3-octanone, no significant differences were found in fresh weight growth as compared to controls (Figure 2B). Mean growth was found to be the highest for doses of 1 µL, which was significantly greater than for doses of 10 µL per 100 g (*p* = 0.016). 

### 3.3. Biostimulatory Effects of M. brunneum, VOCs and Hydrogel Formulations on Four Commercially Important Plant Species

**Figure 3:**
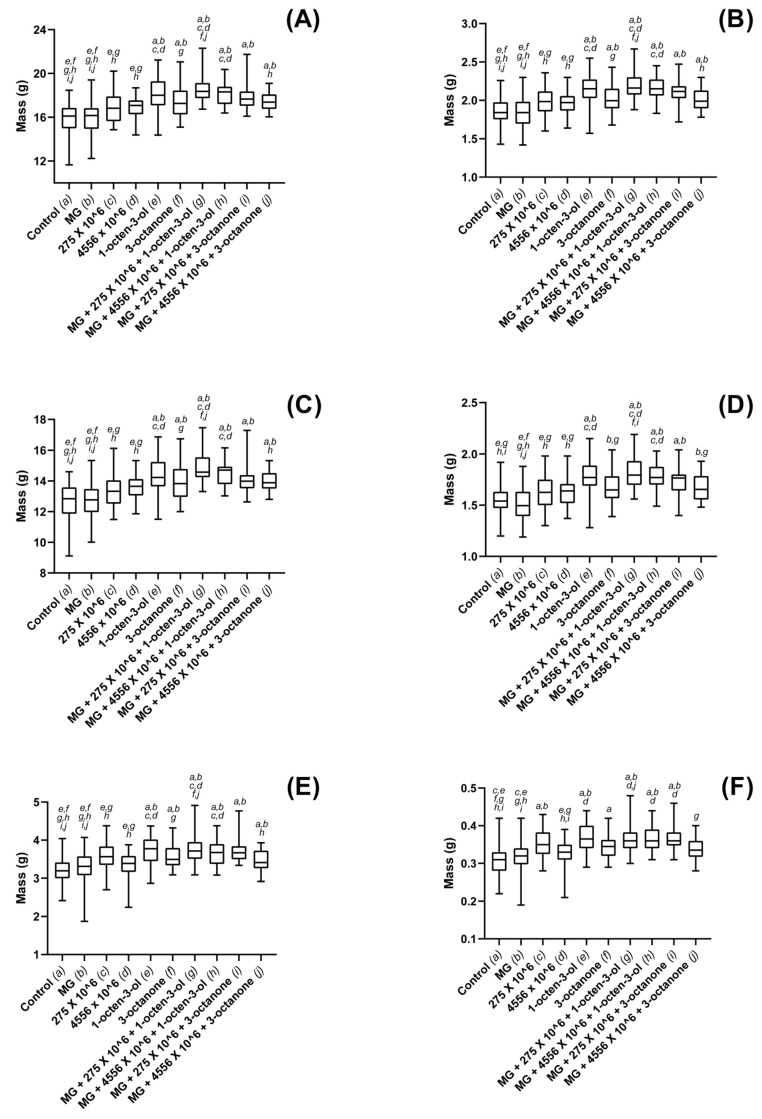
Plant growth metrics for oilseed rape plants exposed to *M. brunneum*, *M. brunneum*-derived VOCs and hydrogels. (**A**) whole-plant fresh mass, (**B**) whole-plant dry mass, (**C**) fresh shoot mass, (**D**) dry shoot mass (**E**) fresh root mass and (**F**) dry root mass, recordings for plants grown in composts treated with hydrogels (MG), *M. brunneum* strains: V275 or 4556, 1-octen-3-ol, 3-octanone or combinations thereof. All *M. brunneum* applications were standardized at 1 × 10^6^ conidia g^−1^ compost, and all VOCs were added at a rate of 1µL 100 g^−1^ compost. Hydrogels were added according to manufacturer specification. Controls were grown in untreated composts. Plants were grown in a glasshouse maintained at 25 ± 2 °C for a total of six weeks. Controls were exposed to compost media without additional treatment. Figure display’s minimum, 1st quartile, median, 3rd quartile and maximum measurements within the treatment groups. Significant results denoted between treatments using letters (*a*–*j*) above each boxplot; where denotation letters are absent, no significant differences were detected between treatments. Key: 275—*M. brunneum* (Strain: V275); 4556—*M. brunneum* (Strain: ARSEF 4556); MG—MiracleGro hydrogel.

Oilseed rape total fresh biomass was significantly larger in treatments containing 1-octen-3-ol (*p* < 0.001) or 3-octanone (*p* < 0.001), when compared to controls (Figure 3A). Plants grown in soils containing 1 × 10^6^ conidia g^−1^ were not found to differ significantly from controls for either V275 (*p* = 0.064) or ARSEF 4556 (*p* = 0.061). Hydrogel-treated soils were also not found to affect plant growth. Combined treatments using *Metarhizium*, VOCs and hydrogels all resulted in significant whole plant weight gains as compared to control (*p* < 0.001). These combination treatments were also found to significantly outperform *Metarhizium* as a sole biostimulant (*p* = 0.001 and 0.003, respectively). Dry total biomass was found to correlate strongly with fresh-weights, and similar significance levels found between treatments (Figure 3B). 

Fresh shoot weights were significantly increased in the presence of both VOCs, as well as all combined treatments, when compared to control treatments (*p* < 0.001) (Figure 3C). In addition, it was found that composts treated with ARSEF 4556 also resulted in significant shoot weight gain over controls (*p* = 0.026), while V275 did not elicit such an effect (*p* = 0.315). Combination treatments containing *Metarhizium* strain V275 and 1-octen-3-ol were the most effective and were found to cause greater growth increases than any treatment containing 3-octanone. Dry mass recordings were of similar significance levels; 1-octen-3-ol as a singular biostimulant or in combination with ARSEF 4556 or V275 was found to significantly improve shoot growth (*p* < 0.001 in all cases). 3-octanone in combination with V275 was also found to elicit significant dry shoot mass increases over controls (*p* < 0.001); however, combinations with ARSEF 4556 did not produce an effect (*p* = 0.107). No other significant effects were noted for dry shoot mass (Figure 3D).

OSR root weights were also significantly increased in all VOC-only and combined treatments (*p* < 0.01) with the sole exception of combinations of ARSEF 4556 and 3-octanone (*p* = 0.135) (Figure 3E). Root weights were also significantly improved in the presence of V275 as an individual treatment (*p* = 0.002). Dry root masses were found to be of consistent significance levels with the controls (Figure 3F). 

**Figure 4:**
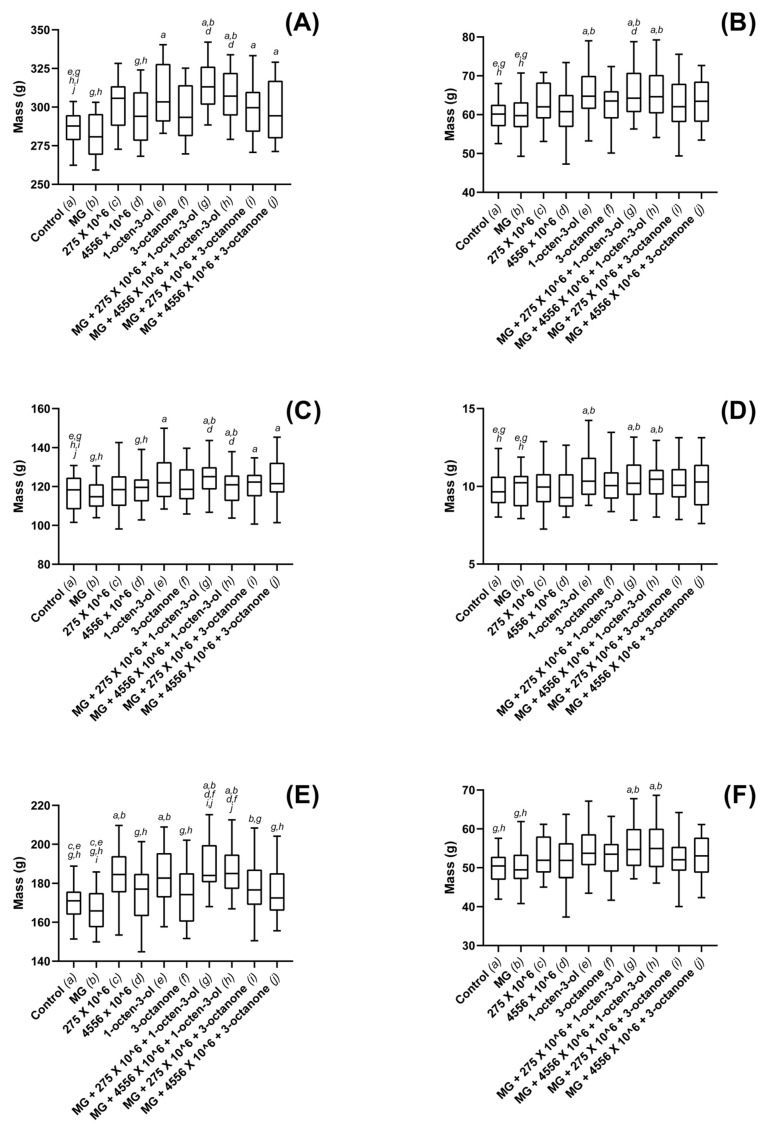
Plant growth metrics for strawberry plants exposed to *M. brunneum*, *M. brunneum*-derived VOCs and hydrogels. (**A**) whole-plant fresh mass, (**B**) whole-plant dry mass, (**C**) fresh shoot mass, (**D**) dry shoot mass (**E**) fresh root mass and (**F**) dry root mass, recordings for plants grown in composts treated with hydrogels (MG), *M. brunneum* strains: V275 or 4556, 1-octen-3-ol, 3-octanone or combinations thereof. All *M. brunneum* applications were standardized at 1 × 10^6^ conidia g^−1^ compost, and all VOCs were added at a rate of 1µL 100 g^−1^ compost. Hydrogels were added according to manufacturer specification. Controls were grown in untreated composts. Plants were grown in a glasshouse maintained at 25 ± 2 °C for a total of six weeks. Controls were exposed to compost media without additional treatment. Figure display’s minimum, 1st quartile, median, 3rd quartile and maximum measurements within the treatment groups. Significant results denoted between treatments using letters (*a*–*j*) above each boxplot; where denotation letters are absent, no significant differences were detected between treatments. Key: 275—*M. brunneum* (Strain: V275); 4556—*M. brunneum* (Strain: ARSEF 4556); MG—MiracleGro hydrogel.

Strawberry plant fresh total biomass was seen to be most affected in the presence of 1-octen-3-ol (*p* < 0.001), either as singular treatment and when applied in combination with ARSEF 4556 (*p* < 0.001) or V275 (*p* < 0.001) (Figure 4A). While treatment with V275 conidia resulted in significant fresh mass gain compared to controls (*p* = 0.015), dry mass recordings for the same plants were not found to differ significantly from controls (*p* = 0.679). Analysis of dry mass otherwise produced similar results to those of the fresh mass recordings, combination treatments of 1-octen-3-ol and V275 producing the greatest biostimulatory effect (Figure 4B). In contrast, shoot mass only consistently increased in treatments combining 1-octen-3-ol with V275 conidia (Figure 4C,D); fresh mass (*p* = 0.033), dry mass (*p* = 0.005). Fresh root biomass increases were seen for many of the treatment groups (Figure 4E). Both V275 (*p* = 0.004) and 1-octen-3-ol (*p* = 0.001) were found to significantly increase root growth as individual treatments. Combinations of 1-octen-3-ol with V275 were found to outperform 3-octanone combinations with both ARSEF 4556 (*p* = 0.002) and V275 (*p* = 0.020), though all combined treatments were found to be more effective than treatment with conidia or VOCs individually (Figure 4E). Dry root mass was also found to have significantly increased for plants treated with combinations of 1-octen-3-ol and ARSEF 4556 (*p* = 0.011) or V275 (*p* = 0.028); however, no other significant differences were detected in dry root masses between treatments and the controls (Figure 4F).

**Figure 5:**
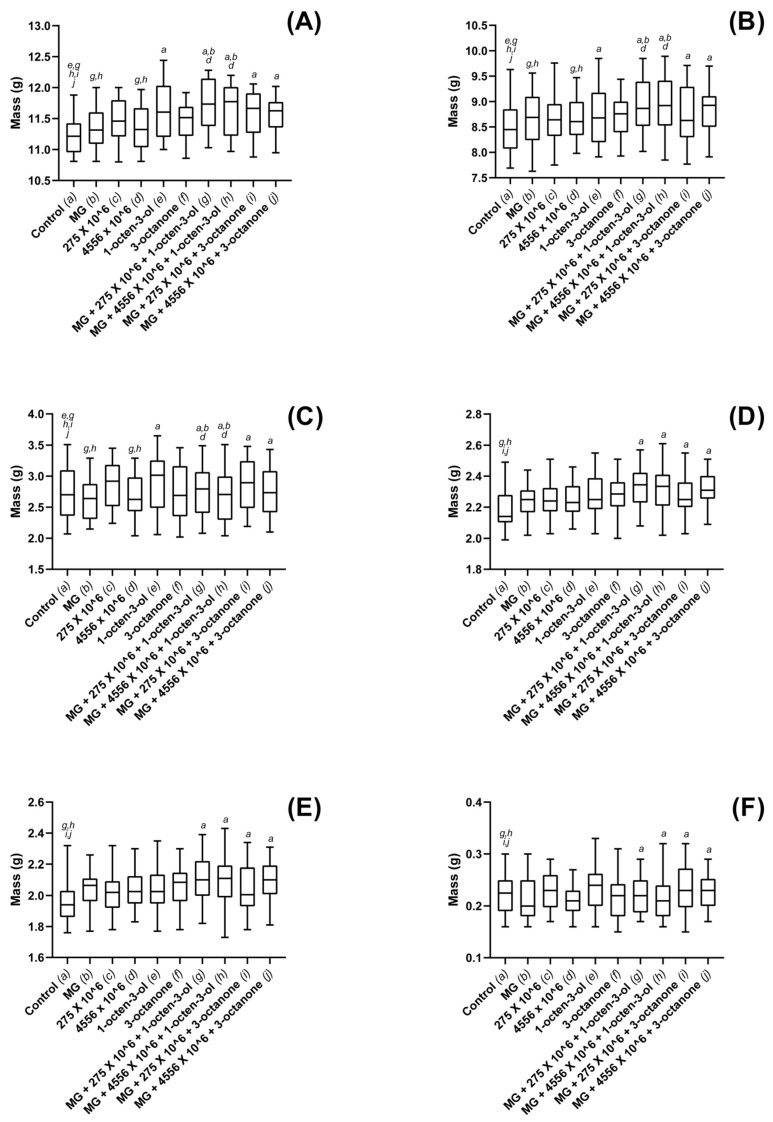
Plant growth metrics for maize plants exposed to *M. brunneum*, *M. brunneum*-derived VOCs and hydrogels. (**A**) whole-plant fresh mass, (**B**) whole-plant dry mass, (**C**) fresh shoot mass, (**D**) dry shoot mass (**E**) fresh root mass and (**F**) dry root mass, recordings for plants grown in composts treated with hydrogels (MG), *M. brunneum* strains: V275 or 4556, 1-octen-3-ol, 3-octanone or combinations thereof. All *M. brunneum* applications were standardized at 1 × 10^6^ conidia g^−1^ compost, and all VOCs were added at a rate of 1µL 100 g^−1^ compost. Hydrogels were added according to manufacturer specification. Controls were grown in untreated composts. Plants were grown in a glasshouse maintained at 25 ± 2 °C for a total of six weeks. Controls were exposed to compost media without additional treatment. Figure display’s minimum, 1st quartile, median, 3rd quartile and maximum measurements within the treatment groups. Significant results denoted between treatments using letters (*a*–*j*) above each boxplot; where denotation letters are absent, no significant differences were detected between treatments. Key: 275—*M. brunneum* (Strain: V275); 4556—*M. brunneum* (Strain: ARSEF 4556); MG—MiracleGro hydrogel.

Maize plant total fresh biomass was significantly increased in all treatments using combinations of 1-octen-3-ol or 3-octanone with *M. brunneum* conidia (Figure 5A). 1-octen-3-ol was also found to be effective as an individual biostimulant (*p* < 0.001). 3-octanone acting as a sole agent did not stimulate significant growth over controls (*p* = 0.317). Dry-mass recordings correlated well with fresh-mass recordings (Figure 5B), combination treatments of 1-octen-3-ol with ARSEF 4556 (*p* = 0.007) and V275 (*p* < 0.001) were the most effective, although treatments using 1-octen-3-ol only were not found to differ significantly to controls (*p* = 0.075), as opposed to fresh weight recordings. In terms of fresh shoot mass, significant increases were found only in combined treatments incorporating 1-octen-3-ol and either ARSEF 4556 (*p* = 0.018) or V275 (*p* = 0.011) (Figure 5C). Dry shoot masses were strongly correlated to fresh mass (Figure 5D); however, significant increases were also found for treatments using combinations of 3-octanone and ARSEF 4556. No significant changes in fresh root mass were recorded in any of the treatments included in the study (Figure 5E); however, dry root mass was found to have increased (Figure 5F) for treatments combining 1-octen-3-ol or 3-octanone with either of the *h* strains (*p* < 0.049).

**Figure 6:**
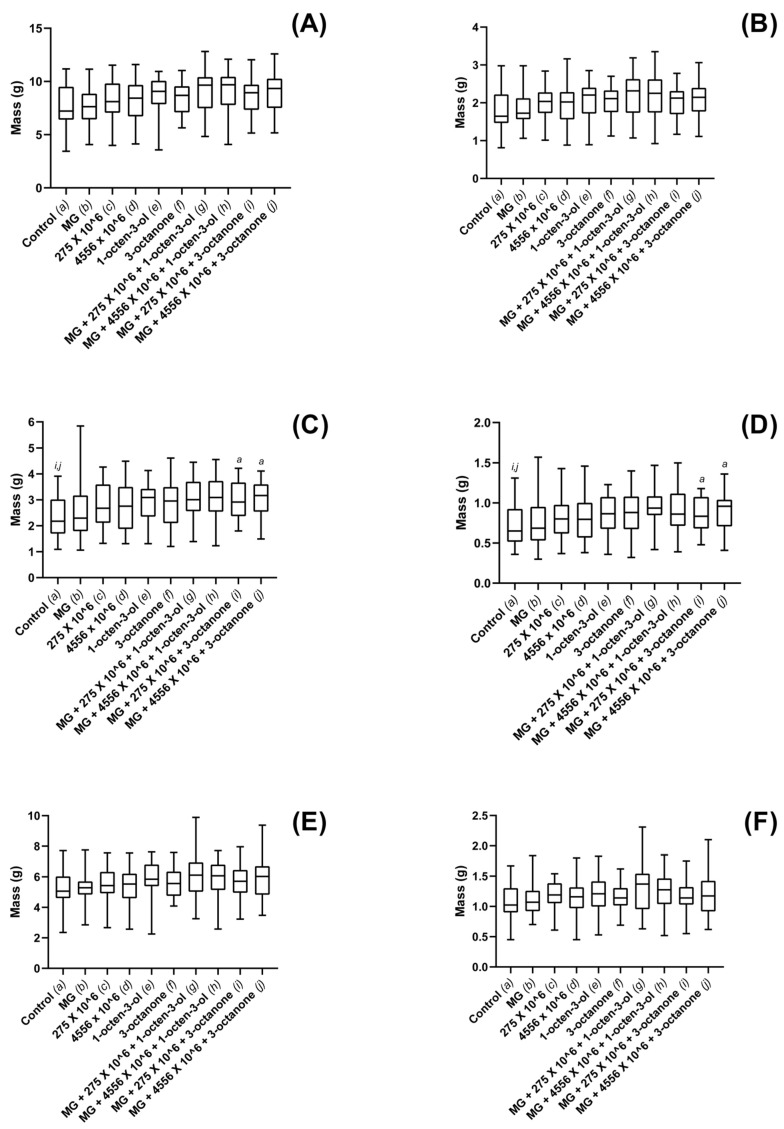
Plant growth metrics for maize plants exposed to *M. brunneum*, *M. brunneum*-derived VOCs and hydrogels. (**A**) whole-plant fresh mass, (**B**) whole-plant dry mass, (**C**) fresh shoot mass, (**D**) dry shoot mass (**E**) fresh root mass and (**F**) dry root mass, recordings for plants grown in composts treated with hydrogels (MG), *M. brunneum* strains: V275 or 4556, 1-octen-3-ol, 3-octanone or combinations thereof. All *M. brunneum* applications were standardized at 1 × 10^6^ conidia g^−1^ compost, and all VOCs were added at a rate of 1µL 100 g^−1^ compost. Hydrogels were added according to manufacturer specification. Controls were grown in untreated composts. Plants were grown in a glasshouse maintained at 25 ± 2 °C for a total of 12 weeks. Controls were exposed to compost media without additional treatment. Figure display’s minimum, 1st quartile, median, 3rd quartile and maximum measurements within the treatment groups. Significant results denoted between treatments using letters (*a*–*j*) above each boxplot; where denotation letters are absent, no significant differences were detected between treatments. Key: 275—*M. brunneum* (Strain: V275); 4556—*M. brunneum* (Strain: ARSEF 4556); MG—MiracleGro hydrogel.

Sitka spruce total biomass was not found to differ significantly between any of the treatments included in the study (Figure 6A,B); however, shoot mass was found to be significantly higher (Figure 6C,D) for combined treatments of 3-octanone and V275 or ARSEF 4556 conidia. Fresh and dried shoot mass was found to be significantly enhanced for those plants treated with combinations of 3-octanone with either V275 (*p* = 0.027) or ARSEF 4556 (0.035) (Figure 6C,D). 1-octen-3-ol combinations with ARSEF 4556 (*p* = 0.058) and V275 (*p* = 0.078) were not found to cause significant shoot growth increases. No individual EPF or VOC treatments were found to cause significant shoot mass increases. Fresh root mass was not found to be affected by any of the treatments (Figure 6E); however, dry root mass was seen to have significantly increased for the combined treatment of 1-octen-3-ol and V275 (*p* = 0.049). No other significant differences were found between treatments (Figure 6F).

## 4. Discussion

Entomopathogenic fungi including *Metarhizium brunneum* are known to enhance parameters of plant growth through a variety of endophytic and rhizosphere activities. The precise mechanisms of microbially mediated plant growth promotion are diverse and have been shown to be largely influenced by the regulation of plant and fungal metabolism [22,23,24]; however, the effect of fungal metabolites as contributory biostimulants has not been investigated. This study demonstrates that not only is *M. brunneum* an effective biostimulant across a diverse range of commercially important plants, but *M. brunneum*-derived VOCs are highly effective plant biostimulants in their own right. 

Baseline assays in this study demonstrate the efficacy of *M. brunneum* conidia as an individual biostimulant at a range of concentrations, supporting the findings of prior research [5,15,41]. Dose efficacy was seen to be a bell-curve response, whereby stimulation was seen to peak at 1 × 10^6^ conidia g^−1^ compost, while at higher concentrations growth was seen to stagnate or decline (Figure 1). Microbial competition within the rhizosphere, as well as within the plant, is fierce [42]. Strong population bias for any given species is likely to lead to declines in total microbial diversity and the differential effects exhibited by endophytes may be attributed to both plant and fungal species and their ability to interact and adapt to the variety of *in planta* conditions, both biotic and abiotic [15,24,43]. It may be that high a concentration of *M. brunneum* conidia within the soil leads to a reduction in other beneficial microbes important for plant development, thereby impacting reducing the biostimulatory effect of the EPF at such concentrations.

Dosage was seen to affect both shoot and root growth when exposed to either strain of the fungus; however, using the optimized concentration, it was seen that larger shoots did not necessarily correlate to larger root systems, and vice versa (Figure 3, Figure 4, Figure 5 and Figure 6). Such disparity suggests that growth of the above- and below-ground portions of the plant are not inextricably linked. Furthermore, given that 4556 appeared generally more efficacious in promoting shoot length, while V275 generally promoted root length to a greater degree, it may be that the biostimulatory effects of EPF are not only species specific, but may in fact differ between strains of the same species. Isolation of the most effective strains for biostimulatory effects will therefore be of paramount importance for future product development and implementation. 

The inclusion of hydrogels into the final experiments demonstrates the compatibility of *M. brunneum* with a commercial hydrogel product, outlining potential for a novel formulation strategy. Synthetic hydrophilic polymers (hydrogels) are a particular class of gels, obtained by chemical stabilization of hydrophilic polymers in a tridimensional network. Hydrogels have been widely utilized within horticulture as their physical properties can be used to mitigate for a lack of water and nutrients in the environment [44,45]. Alongside their conventional purposes, hydrogels have been found useful as application adjuvants for slow-release fertilizers and pesticides [38,39]. While potassium acrylate hydrogels are biodegradable, the process is slow. Development of a hydrogel resourced from natural products, such as those made from starch, that is compatible with *Metarhizium* and other EPF may offer an exciting avenue of formulation that could be used to simplify and enhance biostimulation under horticultural, agricultural and forestry sectors [37].

Entomopathogenic fungi such as *Metarhizium* and *Beauveria* spp. are known to have a broad metabolic repertoire [18,20,46]. Some components of the VOC profile of *M. brunneum* have been characterized, with 1-octen-3-ol and 3-octanone being primary metabolic by-products [29,30], both of which are known metabolites produced by several plant and fungal species [47,48,49]. In relation to *M. brunneum* and other fungi, the primary functions of these compounds are thought to relate to microbial competitor inhibition [28,50]. More recently, however, they have also been shown to act as efficient semiochemicals, insecticides and molluscicides [30,50,51]. While the biostimulatory effects of *Metarhizium* are well documented [52], this is the first study to demonstrate that *Metarhizium*-derived VOCs can significantly improve plant growth independent of microbial activity.

Assays to determine the optimum concentrations of each VOC produced variable results. 1-octen-3-ol was found to be the most effective compound but was more dose sensitive (Figure 2). Inhibitory effects to growth were found at the highest applied doses of the VOC; 33% of the seeds failed to germinate (data not shown) and those that did, grew to only 73% of the size of the controls. While inhibitory effects were seen for the highest doses of 1-octen-3-ol, no negative effects were seen on germination or the production of healthy shoot and root lengths at the lowest doses, and no significant effects were found at all for plants exposed to 3-octanone (Figure 2). Inconsistency between results suggests that dosage will be a key factor in prescribing optimal applications to elicit the desired biostimulatory effects. Given that 3-octanone was not found to elicit phytotoxic effects at any concentration, potentially making it an ideal candidate as a high-concentration pesticide against pest species such as plant-parasitic nematodes [29]. Furthermore, of the compounds characterized as part of the *M. brunneum* VOC bouquet [29], only 20 were screened prior to these studies (data not published). Further elucidation of the full volatile profile of *M. brunneum* may lead to the discovery of new compounds with similar or greater effects. 

Optimized applications of each of the *M. brunneum* conidia and VOCs were taken forward for full assessment in strawberries, maize, OSR and sitka spruce; species representing a spectrum of commercially important grasses, brassicas, soft fruits and trees. Combinations of 1-octen-3-ol and *M. brunneum* were found to be highly effective biostimulants for all plants assayed, for the three angiosperms; maize, OSR and strawberry; 1-octen-3-ol was highly efficacious as an individual biostimulant. In no experiments was the inclusion of hydrogels as a potential EPF carrier found to affect plant growth. The exact mechanisms of plant growth promotion by *Metarhizium* VOCs are still unknown; both direct effects such as upregulation of plant hormones and indirect effects including plant protection properties are plausible contributors. To date, a range of physiological adaptations to microbe-originated VOCs have been recorded. For instance, the first study that demonstrated microbial VOCs as seedling biostimulants showed that volatiles released by *Bacillus subtilis* GB03 elicited a ∼5-fold increase in total leaf area of *A. thaliana* [33]. Plant and animal-derived biostimulant compounds have been found to increase trichoblast production in the root systems of plants [53], thereby increasing total root volume and mass. Such effects may eventually yield larger plant mass via increased acquisition of soil nutrients. Regulation of soil microbiota by such compounds may also confer physiological changes in the plant by altering nutrient uptake or reducing phytophagous microbiota and invertebrates that could lead to damage during growth phases [54]. Several previous studies have demonstrated that microbial VOCs are derived from fungi; *Alternaria alternata* [55] and *Fusarium oxysporum* [56] are also able to increase chlorophyll content and other photosynthetic parameters of plants. It may be that the plants are able to utilize the VOCs, or at least components of them, in order to produce more chlorophyll, or that chlorophyll production is directly influenced by the VOCs, thereby increasing photosynthetic potential. If this should prove to be the case, then it would appear that this increased potential contributes to significantly improved growth. Zhang et al. [57] also demonstrated that exposure of *A. thaliana* plants to *B. subtillis* volatiles resulted in an increase in chlorophyll concentration of 84%, mediated by the activation of the plant’s own iron acquisition machinery to increase assimilation of metal ions. The *Metarhizium*-derived VOCs may also act antagonistically to other damaging microbes [26], thereby indirectly contributing to plant growth through altering rhizosphere biodiversity in favour of the plant.

While the full extent of the complex mechanism for VOC mediated biostimulation remain unclear, this study demonstrates the high efficacy of *Metarhizium*-derived VOCs and biostimulants in a wide range of commercial plants. The compounds were both effective as individual products and as combined actors in conjunction with hydrogels and *M. brunneum* conidia, opening new potential development avenues for the deployment of both plant protection and plant stimulation products. Further investigation may lead to significant developments in our understanding of the chemical ecology exhibited between fungi and plants, alongside the development of novel plant biostimulant compounds that can be produced and applied effectively within agricultural sectors.
